# Universality and chaoticity in ultracold K+KRb chemical reactions

**DOI:** 10.1038/ncomms15897

**Published:** 2017-07-19

**Authors:** J. F. E. Croft, C. Makrides, M. Li, A. Petrov, B. K. Kendrick, N. Balakrishnan, S. Kotochigova

**Affiliations:** 1 Department of Chemistry, University of Nevada, Las Vegas, Nevada 89154, USA; 2 Department of Physics, Temple University, Philadelphia, Pennsylvania 19122, USA; 3 NRC ‘Kurchatov Institute’ PNPI, Gatchina, Leningrad District 188300 Russia; 4 Division of Quantum Mechanics, St Petersburg State University, 7/9 Universitetskaya nab., St Petersburg 199034, Russia; 5 Theoretical Division (T-1, MS B221), Los Alamos National Laboratory, Los Alamos, New Mexico 87545, USA

## Abstract

A fundamental question in the study of chemical reactions is how reactions proceed at a collision energy close to absolute zero. This question is no longer hypothetical: quantum degenerate gases of atoms and molecules can now be created at temperatures lower than a few tens of nanokelvin. Here we consider the benchmark ultracold reaction between, the most-celebrated ultracold molecule, KRb and K. We map out an accurate *ab initio* ground-state potential energy surface of the K_2_Rb complex in full dimensionality and report numerically-exact quantum-mechanical reaction dynamics. The distribution of rotationally resolved rates is shown to be Poissonian. An analysis of the hyperspherical adiabatic potential curves explains this statistical character revealing a chaotic distribution for the short-range collision complex that plays a key role in governing the reaction outcome.

## Abstract (web summary)

Studying chemical reactions near zero temperature in detail is challenging both in theory and practice. Here the authors report an explicit quantum mechanical study of the benchmark ultracold reaction between a K atom and a KRb molecule, important for future controlled chemistry experiments.

The ability to prepare reactants and control products on demand with quantum state precision is chemistry's holy grail^1^,^2^,^3^,^4^,^5^,^6^,^7^,^8^,^9^,^10^. Ultracold chemistry is a new and rapidly progressing field where reactants are prepared in a single quantum state which holds out this promise^1^,^10^,^11^,^12^. In a pioneering experiment, groups at JILA were able to produce an ultracold gas of ^40^K^87^Rb molecules at nano-Kelvin temperature where even the nuclear spins were oriented^1^,^13^. The exothermic reaction rate coefficients of KRb+KRb and KRb+K were also measured. In addition, by merely flipping a nuclear spin the KRb+KRb reaction could be turned on and off. This is a perfect illustration of an on demand reaction demonstrating the control that is attainable in ultracold gases. Such control also promises to answer open questions such as the role of geometric phases (GPs), non-adiabatic^14^ and Van der Waals entrance-channel effects^15^, and the effect of external fields on the product distribution in chemical reactivity. Detection of complex molecules with single-quantum state precision has been shown to be feasible in a hotter 1 K environment^16^,^17^.

However, theoretical calculations of reaction dynamics for such systems pose a daunting computational challenge. Ultracold collisions are sensitive to details of the potential and require accurate electronic potential calculations. In addition many of the systems of current interest are heavy with deep potentials, meaning that scattering calculations need to include many channels with computational costs scaling with the cube of the number of channels. It is for these reasons that while the pioneering experiments^1^,^13^, on KRb reactions, were performed over 6 years ago no accompanying scattering calculations have been performed, until now. Such calculations are needed to provide key predictions for state-to-state rate coefficients, which await laboratory measurement.

The reaction of KRb with K is relatively fast with a rate coefficient of the order of 10^−10^ cm^3^ s^−1^ which agrees fairly well with predictions of a universal model (UM), based on quantum defect theory^18^,^19^. This suggests that most molecules undergo atom exchange, and only a small percentage undergo an elastic collision. A statistical approach has been used to analyse the effect of an external electric field on the rotational product distribution in the K+Rb_2_ and KRb+KRb reactions^20^. However, to understand the chemistry of such reactions one needs to go beyond these approaches and examine reaction rates at a state-to-state level.

The reactive scattering of complex atoms and molecules has always been intricately intertwined with classical and quantum chaos. In particular, classical trajectory simulations of reactive scattering show self-similar behaviour of dwell times between entering and leaving the central part of the high-dimensional potential^21^,^22^,^23^. Recently there has been interest in the chaotic character of ultracold inelastic collisions^24^,^25^,^26^,^27^. Atom+dimer collisions involving three identical alkali atoms at ultracold energies have been shown to be classically chaotic^28^. This chaotic character has been taken as the starting point for work examining statistical aspects of non-reactive ultracold alkali-metal dimer collisions^29^,^30^ and ultracold resonance reactions^31^. Such works suggest an approach to tackling ultracold reactions involving heavy alkali species avoiding the prohibitive computational cost of numerically exact calculations.

We report an explicit quantum mechanical study of the ultracold reaction between a K atom and a KRb molecule. To this end, we compute an accurate *ab initio* ground-state potential energy surface (PES) of the KRbK complex in full dimensionality, taking special care to accurately describe the long-range forces important in ultracold collisions. The total rate is shown to be universal in character—validating the use of simple UMs for other similar reactions. On the other hand, the product rotational distribution is shown to be statistical in character, which we attribute to the chaotic nature of the reaction complex.

## Results

### Potential energy surface calculation

The first theoretical studies of the electronic structure of three- and four-body alkali-metal systems focused on homonuclear trimers^32^,^33^,^34^. Initial studies of heteronuclear alkali-metal trimer and tetramer potentials principally located optimized geometries and dissociation energies^35^,^36^,^37^.

In an alkali-metal trimer the three valence electrons, one from each atom, couple during the reaction and create two doublet and one quartet adiabatic potential surfaces^35^. The energetically lowest is a doublet ^2^*A*′ potential. We have studied the reaction dynamics along this potential surface and it is the focus of our electronic structure calculation.

Complete and accurate information on the lowest KRbK potential surfaces is unavailable. Their computation requires substantial effort due to the complexity of the multi-electron and open-shell reactants. In this work, we perform a systematic *ab initio* study using the multi-reference configuration-interaction (MRCI) method of the chemistry package MOLPRO^38^. Details of this calculation can be found in ‘Methods’.

**Figure 1:**
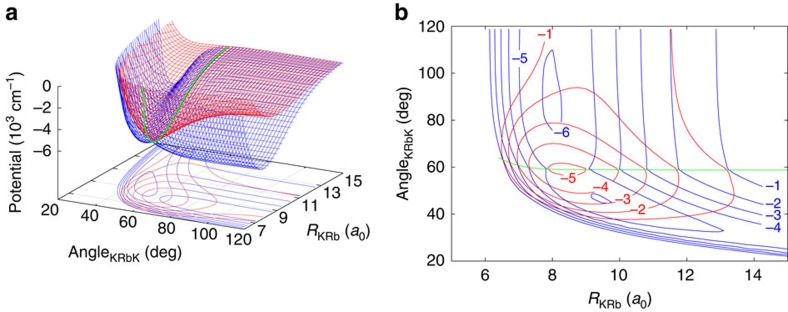
K–KRb potential energy surface. (**a**) A two-dimensional cut through the energetically lowest ^2^*A*′ (blue curve) and ^2^*B*′ (red curve) adiabatic potential energy surfaces of the KRbK trimer as a function of the K−Rb bond lengths and the angle between K−Rb−K along the isosceles *C*_2*ν*_ geometry with *R*_K1Rb_=*R*_K2Rb_. The base of the figure shows the corresponding contour graph. The zero of energy corresponds to the energy of three well-separated atoms. The unit of length is the Bohr radius *a*_0_=0.0529177, nm and that of energy is wavenumbers. (**b**) A contour graph of the K−Rb−K potentials based on the data in **a**. Contour labels are in units of 10^3^ cm^−1^. In both panels the seam of conical intersections is shown by the green line.

Figure 1a shows a two-dimensional (2D) cut of the energetically lowest adiabatic potentials, ^2^*A*′ and 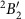, along the isosceles *C*_2*v*_ geometry where *R*_K(1)Rb_=*R*_K(2)Rb_. The reactant and product states are situated in the pairwise potential wells when either *R*_K(1)K(2)_ or *R*_KRb_ is large. We find that the lower ^2^*A*′ potential has an absolute minimum when *R*_K(1)Rb_=11.10*a*_0_, *R*_K(2)Rb_=8.04*a*_0_ and *R*_K(1)K(2)_=7.64*a*_0_. The atomization energy, the minimum energy of three seperated atoms measured from the potential minimum, is *V*_A_=6,258 cm^−1^. The dissociation energy from the optimized geometry and the limit KRb+K is *V*_d1_=2,079 cm^−1^, while that to the limit K_2_+Rb is *V*_d2_=1,854 cm^−1^.

The accuracy of our *ab initio* trimer potential is tested by calculating the *ab initio* dimer X^1^Σ^+^ and a^3^Σ^+^ potentials for both KRb and K_2_ at the same level of electronic-structure theory as the trimer potential. These potentials are compared with their corresponding spectroscopically accurate dimer potentials for KRb^39^ and K_2_ (ref. 40) in ‘Methods’. As discussed in ‘Methods’ comparison between our theory and experiment shows an excellent agreement. In addition, we use the dimer X^1^Σ^+^ and a^3^Σ^+^ potentials to construct the lowest pairwise trimer potentials for KRbK following the dimer-in-molecule theory of ref. 41. Details of the analytical construction of these potentials are given in ‘Methods’.

### Quantum dynamics calculations

**Figure 2:**
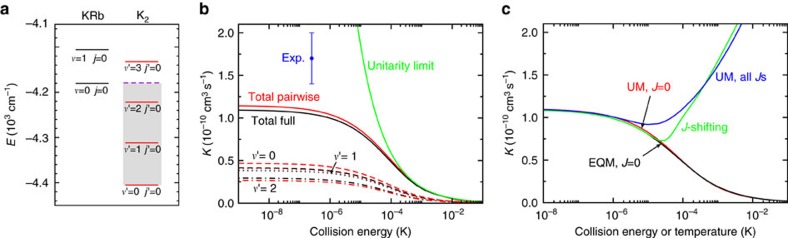
Energetics and reaction rates. (**a**) Energetics of the KRb+K→K_2_+Rb reaction. The *j*=0 vibrational levels of the KRb X^1^Σ^+^ potential and *j*′=0 vibrational levels of the K_2_ 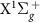 potential are shown by black and red lines, respectively. The grey shaded area indicates the closely spaced energetically allowed rotational levels of K_2_. The zero of energy is located at the dissociation limit of KRb and K_2_. (**b**) Reaction rate coefficients from *J*=0 EQM calculations based on either the full (black curves) or pairwise (red curves) potential as a function of collision energy in units of the Boltzmann constant. The total and vibrationally resolved reaction rate coefficients are shown. The green curve is the *s*-wave unitary rate coefficient for atom-dimer scattering. The closed circle with error bars (one s.d.) corresponds to an experimental measurement^1^ taken at a temperature of 250 nK. (**c**) Total reaction rate coefficient (green curve) as a function of temperature based on the *J*-shifting method and the *J*=0 EQM results for the full trimer potential. The black curve repeats this latter curve from (**b**) as a function of energy. The red curve shows the rate coefficient of the *s*-wave universal model (UM) as a function of collision energy, while the blue curve shows the UM data including all relevant partial waves as a function of temperature.

We present exact quantum-mechanical (EQM) calculations for the KRb(^1^Σ^+^, *v*=0, *j*=0)+K(^2^S)→K_2_(^1^Σ_*g*_^+^, *v*′, *j*′)+Rb(^2^S) chemical reaction, where *v*, *v*′ and *j*, *j*′ are vibrational and rotational quantum numbers, respectively. The lowest ro-vibrational state of the product K_2_ molecule lies Δ/(*hc*)=237 cm^−1^ beneath that of the reactant KRb molecule (Fig. 2a). Collisions can produce K_2_ molecules with *v*′ up to 2 in a multitude of rotational states *j*′ (up to 63 for *v*′=0; 49 for *v*′=1; 28 for *v*′=2). We omit coupling of the orbital angular momenta with both the electron and nuclear spins. Moreover, our results are restricted to total angular momentum *J*=0. Fortunately, we can still compare directly with experimental results in the ultracold regime as only *s*-wave collisions contribute (that is, only *J*=0 is required for KRb in the ground rotational state *j*=0). For non-zero *J*, the computational cost scales prohibitively as 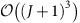 even when we take advantage of parity and exchange symmetries.

We use the atom–diatom scattering formalism developed by Pack and Parker^42^,^43^. In the short-range region we use adiabatically adjusting principle-axis hyperspherical coordinates (APH), an approach which ensures that all atom–diatom arrangements are treated equivalently, while in the long-range region we use Delves hyperspherical coordinates (DC) where a molecular basis is more appropriate. For details see the ‘Methods’ section and our recent application of this approach to the ultracold reactive scattering of LiYb molecule with Li atom^44^

In this article, we focus on state-to-state reaction rate coefficients *K*_*v*′*j*′_(*E*) as functions of the collision energy *E*, *v*′-resolved rate coefficients 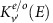 obtained by summing over even and odd product rotational levels *j*′ and, finally, the total rate coefficient 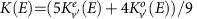 which accounts for the unresolved nuclear spin degeneracies of ^40^K_2_ (ref. 44).

Figure 2b shows the *J*=0 vibrationally resolved and total reaction rate coefficients as a function of the collision energy *E*, along with the experimental total rate coefficient of Ospelkaus *et al*.^1^. Both data for the full trimer potential and that for the pairwise potential are shown. The differences are seen to be small. The rate coefficients reach the Wigner-threshold regime for energies less than a μK and drop off sharply for larger kinetic energies. The drop-off is consistent with the unitarity limit, *v*_*r*_*π*/*k*_*r*_^2^, for a single-entrance partial wave between KRb and K. Here, the relative velocity *v*_*r*_ and wavevector *k*_*r*_ are defined by 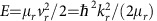 with an atom-dimer reduced mass *μ*_*r*_. This rate coefficient is the absolute upper bound to any ultra-cold reactive process. The vibrationally resolved rate coefficients have the same functional form as the total rate coefficient. The most deeply bound *v*′=0 is most populated followed by *v*′=1 and *v*′=2. The experimental result was obtained at a temperature of 250 nK and is seen to be around 50% larger than ours. We note that our calculations do not include all the effects present in the experiment such as magnetic field, spin and conical intersection effects. We attribute the difference between our calculation and the experiment to these effects. For this system the conical intersection is submerged and lies below the asymptotic energy of the entrance channel, as such the usual vector potential approach for including the GP cannot be used as the singularity at the intersection is exposed. Therefore to include the GP effect the explicit inclusion of the excited doublet surface is necessary.

Figure 2c shows the thermally averaged reactive rate coefficient for the full trimer potential surface as a function of temperature up to 0.1 K evaluated using the *J*-shifting approach^45^. The rate coefficient is seen to have a minimum near *T*=30 μK and slowly increases for larger temperatures. For comparison the non-thermalized EQM results for *J*=0 are repeated, and we emphasize that thermalization does not affect the rate coefficient in the Wigner-threshold regime.

An EQM calculation does not easily lend itself to an intuitive understanding of the collision. To help explain the total reaction rate coefficient we have performed a KRb+K UM calculation^18^,^19^, which only relies on the long-range dispersion coefficient *C*_6_ between the molecule and the atom and the assumption that no flux is returned from short-range separations. For KRb in the *v*=0, *j*=0 ro-vibrational state colliding with K this *C*_6_ was previously determined to be 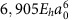 (ref. 19), where *E*_*h*_ is the Hartree energy.

Figure 2c shows the UM rate coefficient as a function of collision energy assuming only *s*-wave collisions as well as that obtained after summing over all relative partial waves. The excellent agreement of the UM with the *J*=0 EQM result and the *J*-shifting method validates the approximations contained in the model and tells us that this reaction occurs with unit probability that is, all flux which reaches the short-range reacts.

**Figure 3:**
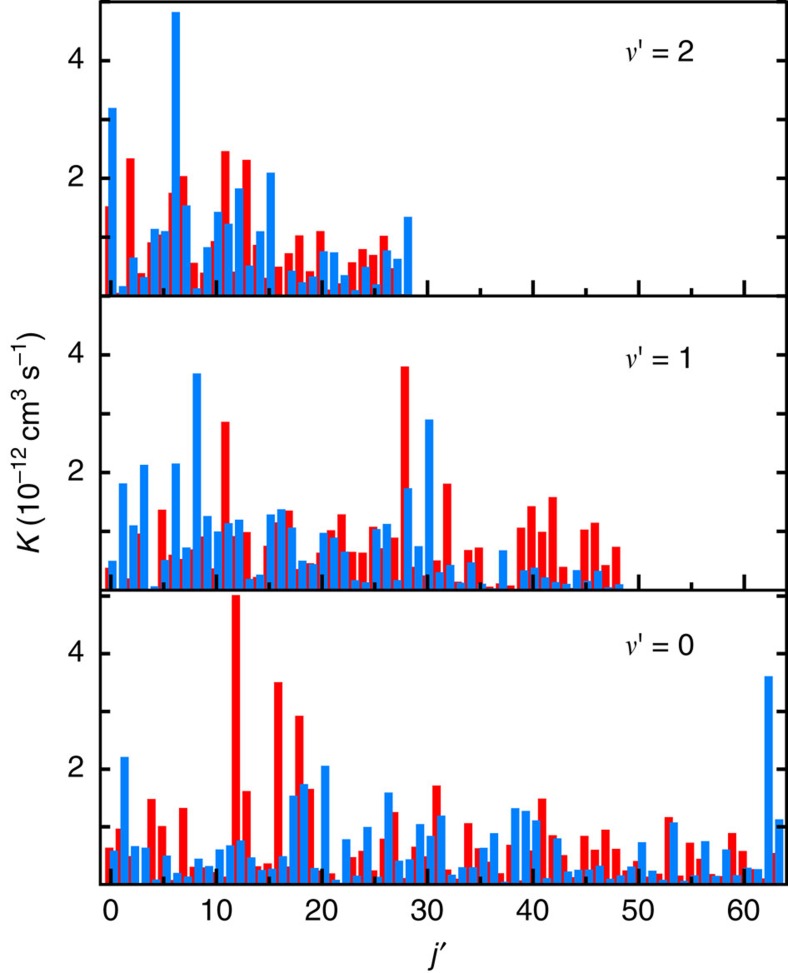
Rotationally resolved rates. The state-to-state *J*=0 EQM reaction rate coefficients from the *v*=0, *j*=0 ro-vibrational state of KRb to the *v*′=0, 1 and 2 vibrational level of K_2_ as a function of product rotational quantum number *j*′. The blue and red bars are from calculations with the full trimer and additive pairwise potential, respectively. Rates are for an initial collision energy of *E*/*k*=210 nK.

Controlled chemistry will require the development of experimental techniques to tune state-to-state rates with an external parameter, such as an electric or magnetic field. As a first step in this direction we compute product *j*′-resolved rates as a function of collision energy. EQM rate coefficients for *J*=0 at *E*/*k*=210 nK are shown in Fig. 3 for both the full and the pairwise potential. The rate coefficients do not vary in the threshold regime and are thus valid for energies below about 1 μK. The *j*′-resolved rates show little obvious structure for either full or pairwise trimer potentials and are uncorrelated even though the *v*′ resolved and total rate coefficients of Fig. 2b for these potentials differ only slightly.

**Figure 4:**
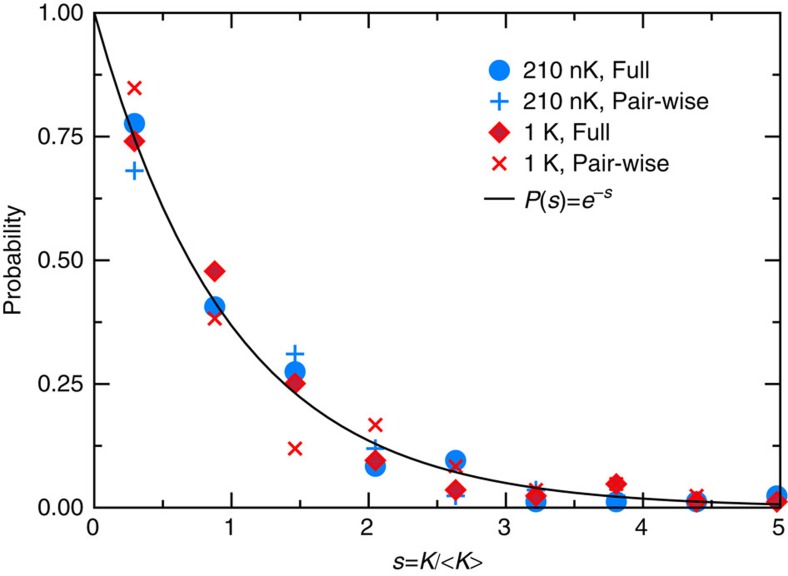
Probability distribution of rotationally resolved rates. Distribution of the *j*′-resolved rate coefficient. Blue and red markers correspond to *J*=0 EQM data populating the *v*′=0, 1 and 2 of K_2_ at initial collision energy *E*/*k*=210 nK and 1 K, respectively. Results for full and pairwise trimer potentials are shown by different markers. For each collision energy the rate coefficients are scaled to its mean value. The solid black curve is the Poisson distribution.

Given the apparent lack of structure seen in the *j*′-resolved rates the question is: how much is it possible to say about these rates? The answer is to think about them statistically. Figure 4 presents the data of Fig. 3 in a different light. We plot the normalized probability distribution of the rotationally resolved rate coefficients *K*_*v*′*j*′_(*E*) for two collision energies and for both the full and pairwise trimer potential. Each distribution is obtained by binning the *K*_*v*′*j*′_(*E*) values into nine equally sized bins, up to five times the mean rate coefficient. Rate coefficients for the three *v*′=0, 1 and 2 vibrational levels are combined to improve the accuracy of the statistical analysis. What in Fig. 3 appeared structureless can now be understood as random variables sampled from the Poisson distribution. We note that the Poisson distribution observed here persists over the entire energy range studied (up to 1 K).

### Chaos and disorder in reactive scattering

What is it about reactions of KRb with K which cause this statistical behaviour? We begin by analysing the adiabatic potentials in the hyperspherical coordinate system for various values of hyper radius *ρ* (computed from our EQM dynamics calculations). The appeal of this approach is that it allows us to visualize and compare the short- and long-range interactions to understand their effect on the reaction dynamics^46^,^47^,^48^.

In the asymptotic limit *ρ*→∞ the adiabatic potentials converge to the reactant and product states, whereas for small *ρ* the shape of the potentials is defined by the ‘variation of the angular potential profile’^49^. For KRbK we include 2,543 such curves, for each symmetry, which consists of all channels that are open at a 220 nK collision energy and those closed channels that are required to converge the reaction rate. For comparison we perform the same analysis for the molecular complex LiYbLi formed during the ultracold reaction LiYb+Li→Li_2_+Yb, studied previously by some of us^44^. The principle difference between these systems is the density-of-states (DOS) which is much smaller for LiYbLi where only 949 states for each symmetry are needed for convergence.

**Figure 5:**
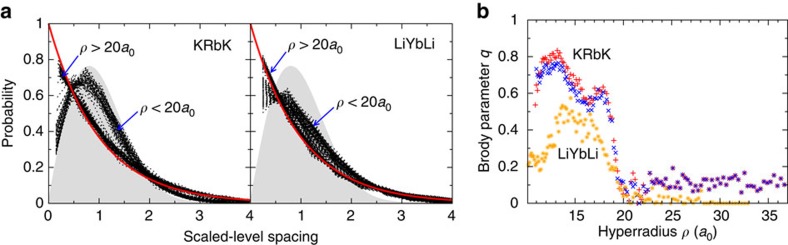
Statistical analysis of the short-range adiabatic potentials. (**a**) Distribution of nearest-neighbor spacings of *J*=0 KRbK and LiYbLi adiabatic potential energies in hyperspherical coordinates. Each black, dotted curve corresponds to the distribution for a single hyper radius *ρ* as a function of scaled level spacing. The shaded grey area and red curve are Wigner–Dyson and Poisson distributions, respectively. For both KRbK and LiYbLi we use the full trimer potential. (**b**) The Brody parameter *q* as a function of hyper radius for both KRbK and LiYbLi as derived from the data in (**a**) for the full trimer potential and data for KRbK based on the pair-wise potential. Blue and red markers correspond to distributions for KRbK obtained with the full and pairwise electronic potentials, respectively, whereas the orange markers for LiYbLi are obtained with the full potential. For *q*→0 the distribution approaches the Poisson distribution while for *q*→1 it approaches the Wigner–Dyson distribution.

We analyse the adiabatic energy level distribution using random matrix theory with the goal to reveal collective behaviour of complex tri-atomic systems. Figure 5a shows nearest-neighbor level-spacing distributions for KRbK and LiYbLi as a function of scaled spacing between the levels. Each curve corresponds to the normalized distribution for one hyperradius and spacings are scaled (divided by the mean spacing). For KRbK we can clearly identify two general trends in the distributions depending on the value of the hyper radius *ρ*. For *ρ*<20*a*_0_, defining the collisional complex, the adiabatic energy levels exhibit Wigner–Dyson behaviour characteristic of quantum chaos, due to the strong interactions. The situation markedly changes at larger *ρ*, where reactants and products of the reaction are energetically well separated. Here, the distribution is described by regular Poisson-like behaviour and statistical disorder. Figure 5a also shows the statistics of the adiabatic energy levels for another trimer complex, LiYbLi. A comparison between the two systems shows that the chaotic regime in the inner region is more pronounced in KRbK than for LiYbLi and we attribute this to the much higher DOS of KRbK.

Figure 5b presents the Brody parameter *q*∈[0, 1] as a function of hyperradius for both KRbK and LiYbLi. It is obtained from fits of 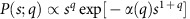 with scaled level spacing *s* (ref. 50) to the data in Fig. 5a. For *q*→0 and 1, the distribution approaches the Poisson and Wigner–Dyson distribution respectively. We see that for both molecules at large *ρ*>20*a*_0_, where the reactant and product molecular levels are well separated and do not repel each other, the Brody parameter *q* has a tendency towards the Poisson distribution. For smaller *ρ*, the Brody parameter becomes much larger for both systems, although for LiYbLi it is always smaller than that for KRbK, consistent with the curves in panel a. The maximum Brody parameter is *q*_max_=0.85 for KRbK and 0.55 for LiYbLi. We identify the chaotic character of the energy levels at short range in KRbK as the cause of the statistical nature of the *j*-resolved rates discussed earlier. The large Brody parameter indicates that many strongly interacting channels contribute to the scattering process. It is the complex interference between these channels which then leads to the Poisson distribution of *j*-resolved rates. The maxima of *q* versus *ρ* near *ρ*=13*a*_0_ and 18*a*_0_ for the KRbK curves correspond to hyperradii where the energetically lowest adiabatic hyperspherical potential has its global and a local minimum, respectively. Finally, we note that even though the effect of the non-additive three-body term in the KRbK potential on the distribution is small, it is large enough to change the product rotational distribution. This is discussed further in the next section.

## Discussion

We have performed numerically-exact quantum mechanical calculations for the reactive collisions of a K atom with a KRb molecule in the ultracold regime. Such calculations are prohibitively expensive, even when neglecting spin and magnetic field effects, especially for heavy alkali systems such as this. We therefore offer these results as a benchmark for future method development desperately needed to guide controlled chemistry techniques.

In addition to these calculations we have applied universal quantum-defect theory to better understand the collisional physics of the reaction forming K_2_ molecules. We find that the reactive rate coefficients of both exact and universal approaches agree well, suggesting the universal character of the reaction and confirm predictions of ref. 1 for this system. The agreement between our numerically exact theory and measurement of ref. 1 is good. We attribute the difference to effects not included in our calculations such as: magnetic field, spin and conical intersection effects.

We found that the role of the non-additive three-body contribution to the potential and to the total reactive scattering of KRbK is small. This confirms our initial conclusion that the total reaction rate coefficient does not depend on details of short-range chemical interactions and is only defined by the long-range interaction properties. We present this as evidence for the validity of UM models when applied to heavy alkali atom+diatom reactions in general.

We have also presented the first prediction of ro-vibrationally resolved reaction rates for this system. We show that *j*-resolved rates can be understood as random variables sampled from a Poisson distribution. The cause of this statistical behaviour is the chaotic nature of complex tri-atomic systems which we quantify with the Brody parameter. We see that the rotational distributions obtained with the full and pairwise potentials are completely different, though both still well described by a Poisson distribution. As this distribution is merely a consequence of the chaotic nature of the complex at short hyperradius this is not specific to reactions of K with KRb. We predict that all ultracold atom+dimer and dimer+dimer reactions involving heavy alkali atoms will exhibit this same Poisson distribution of *j*-resolved rates, provided there are sufficient product channels to perform a statistical analysis.

Our future plans involve a full treatment of the conical intersection and accompanying GP, by including the excited doublet state in the calculations. The GP effect on chemical reactions is well studied, but is often masked by thermal averaging^51^. There is no thermal averaging in the ultracold domain, where the GP has been predicted to have a significant effect^52^,^53^. We also plan to investigate if the system exhibits a quantum butterfly effect where tiny perturbations in the interaction potential lead to exponentially different results (suggested by the completely different *j*-resolved rates seen when the three body term in the potential is included or not). The presence of chaos in such systems would have important implications for the development of controlled chemistry. Studies have shown that small carefully chosen external perturbations can produce a large beneficial change in the system behaviour^54^,^55^,^56^.

We have shown that ultracold reactions of heavy alkali systems are inherently statistical in nature. We believe that this has significant implications for the development of controlled chemistry as well as suggesting a way forward in attacking the currently intractable computational challenge such systems pose.

## Methods

### Non-additive full trimer potential

We have computed the ground-state three-dimensional surface 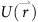 for KRbK using the MRCI method within the MOLPRO software package, where 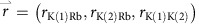 and *r*_K(*i*)R*b*_ are the separations between the *i*-th K atom and Rb and *r*_K(1)*K*(2)_ is the separation between the two K atoms. For the closed shell electrons of K and Rb we employed the ECP18SDF and ECP36SDF energy-consistent, single-valence electron, relativistic pseudo-potentials of the Stuttgart/Cologne groups^57^,^58^. Core polarization potentials are modelled after ref. 59 with cutoff functions with exponents 0.265 and 0.36 for Rb and K, respectively. So each atom is described by a single electron model. We used an uncontracted *sp* basis set supplemented with ECP core potentials and augmented by additional *s*, *p*, *d* and *f* functions^35^.

The *ab initio* electronic structure computations are too expensive to be used for each geometry of the collisional complex. Consequently, following^60^ our discrete data on the three-body contribution (with the pairwise contribution removed) is fitted to a suitable analytical functional form, where adjustable parameters are found with a least-squares procedure. We find that the fit reproduces the PES without introducing spurious features.

**Figure 6:**
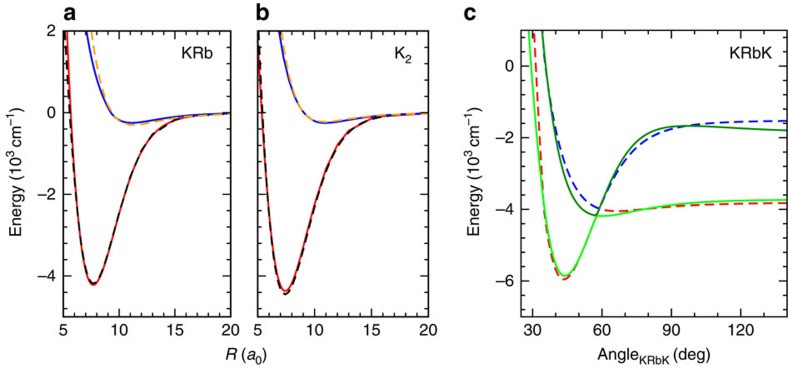
Dimer and trimer potential energy curves. The calculated singlet X^1^Σ^+^ and triplet a^3^Σ^+^ potentials of KRb (solid lines in **a**) and K_2_ (solid lines in **b**) as a function of inter-atomic separation using the same basis set and core polarization potential as for the KRbK trimer. The dashed lines in both panels are the corresponding spectroscopically accurate dimer potentials^39^,^40^. (**c**) A one-dimensional cut through the two energetically lowest adiabatic KRbK trimer potential surfaces based on full (solid lines) and pairwise (dashed lines) calculations for *R*_K(1)Rb_=*R*_K(2)Rb_=10*a*_0_.

We also computed the *ab initio* dimer X^1^Σ^+^ and a^3^Σ^+^ potentials for KRb and K_2_ at the same level of electronic-structure theory as the trimer potential. These dimer potentials are shown in Fig. 6a,b and compared with spectroscopically accurate potentials of KRb^39^ and K_2_ (ref. 40). Agreement is better than 38 cm^−1^ for KRb and 83 cm^−1^ for K_2_ at the equilibrium separation. The comparison of these potential curves provides evidence that the non-additive term in KRbK is small.

**Figure 7:**
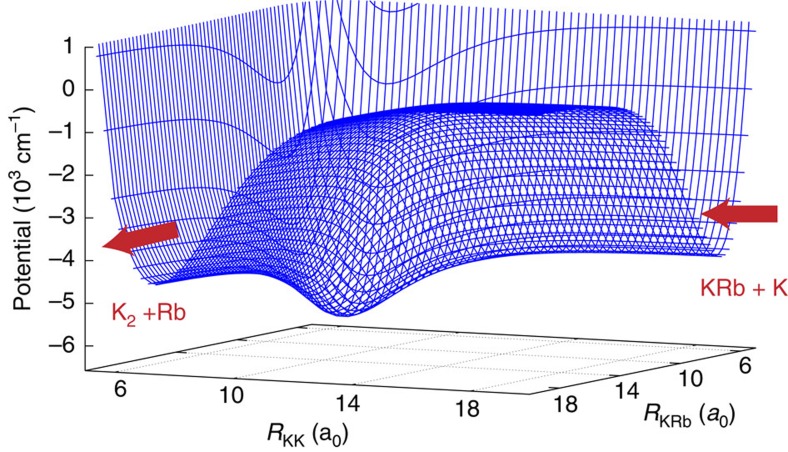
K–KRb potential energy surface for collinear geometry. A two-dimensional cut through the energetically lowest ^2^*A*′ adiabatic potential energy surface of the KRbK trimer as a function of the K−Rb and K−K bond lengths along the collinear geometry. The zero of energy corresponds to the energy of three well-separated atoms. The arrows indicate the entrance and exit channels of the chemical reaction.

Figure 7 shows a 2D cut through the energetically lowest ^2^*A*′ adiabatic potential energy surface of the KRbK trimer as a function of the K−Rb and K−K bond lengths for the collinear geometry. This figure clearly shows that the potential of the collisional complex, where all atoms are close together, is deeper than that of the reactant and product configurations with atoms spending some time moving within the complex before reacting and flying out as K_2_+Rb.

### Pairwise trimer potential

For our coupled-channels calculation we have also used a doublet trimer potential constructed from the pairwise singlet 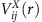 and triplet 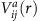 potentials. Here indices *i* and *j* label the atoms and *r* is their separation. The wavefunction of ground-state alkali-metal atoms is uniquely described by its electron spin *s*_*i*_=1/2, where *i*=A, B or C. Two such atoms couple to spin states 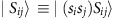, with a total spin singlet 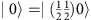 and triplet 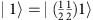 state. Their pairwise potential Hamiltonian is denoted by 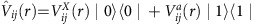. Three atoms couple to states |(*s*_A_*s*_B_)*S*_AB_, *s*_C_; *S*〉. For a doublet total electron spin of *S*=1/2 there exist two ways to couple the spins. These are 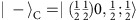 and 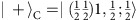, where subscript C in |±〉_C_ indicates that first the spins of atoms A and B are coupled together to a total spin *S*_AB_, which is then coupled to that of atom C. The pairwise trimer potential 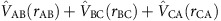 leads to a 2 × 2 matrix in the |±〉_C_ basis. It is most-easily constructed by using angular momentum algebra to transfer between the equivalent basis sets |±〉_A_, |±〉_B_, and |±〉_C_. Diagonalization of this Hamiltonian matrix leads to the two adiabatic ^2^*A* potentials


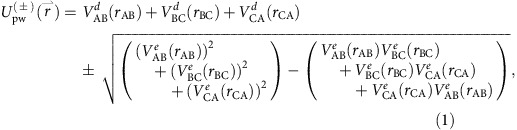


where the dispersion (*d*) and exchange (*e*) potential are


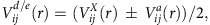


respectively. For our KRbK trimer A=K(1), B=Rb and C=K(2). By construction the factor in the square root is non-negative. Hence, potential 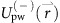 has the lowest energy and is used in our EQM calculations. As an aside we note that in the *C*_2*v*_ symmetry the factor in the square root can be zero, the two potentials are degenerate and the system has conical intersections.

### Exact quantum dynamics calculations

For details of the EQM method see our previous paper^44^ on LiYbLi and references therein, which also contains details of the calculation of the LiYbLi adiabatic potential energies used in this work. Here we outline details specific to the KRbK calculation.

The EQM calculations can broadly be split into three main steps: the numerical computation of 5D hyperspherical surface functions in the APH coordinates in the short-range region and DC in the long-range region; the log-derivative propagation of the CC equation in these coordinates; finally, the asymptotic matching to ro-vibrational states in Jacobi coordinates.

The 5D APH surface functions in the short-range region, from *ρ*=8.0*a*_0_ to 38.15*a*_0_, are functions of two internal coordinates *θ* and *ϕ* and three Euler angles *α*, *β* and *γ* to orient the molecule in space. APH surface functions in *θ* and ϕ are determined by *l*_max_ and *m*_max_ respectively. This region is further subdivided into six, with increasing *l*_max_ and *m*_max_ to ensure converged surface functions. These regions are 8.0*a*_0_<*ρ*<11.33*a*_0_, 11.33*a*_0_<*ρ*<16.87*a*_0_, 16.87*a*_0_<*ρ*<18.82*a*_0_, 18.82*a*_0_<*ρ*<22.74*a*_0_ and 22.74*a*_0_<*ρ*<32.21*a*_0_ with *l*_max_=103, 117, 123, 149, 169, 189 and *m*_max_=206, 234, 246, 298, 338, 378, respectively. For *J*=0 this leads to 5D surface function matrices of dimension 42,952; 55,342; 61,132; 89,550; 115,090; and 143,830. Explicit diagonalization of such large matrices is not computationally tractable so we use the sequential diagonalization truncation technique^61^,^62^ to reduce the dimension and the implicitly restarted lanczos method^63^ to compute only the lowest 2,543 surface functions of each exchange symmetry needed for the log-derivative propagation. These surface functions are computed on a logarithmic grid in *ρ* with 157 sectors.

Delves coordinates are used in the outer region from *ρ*=32.21*a*_0_ to *ρ*_max_=174.95*a*_0_ where a linear grid in *ρ* is used with 456 sectors. The number of basis functions is determined by an energy cutoff of 0.3 eV relative to the minimum energy of the asymptotic K_2_ diatomic potential. A one-dimensional Numerov method is used to compute the adiabatic surface functions.

The log-derivative matrix is propagated separately for each exchange symmetry and parity, though only even parity is needed in this work as *J*=0. In the APH region the propagation includes 2,543 channels of each symmetry, over 20% of which are closed at all *ρ*. The DC region only requires 423 for each symmetry as many of the channels locally open at short range have become strongly closed. Consequently the long-range propagation takes a negligible time compared to the short-range as the computational cost scales as the number of channels cubed.

At *ρ*=*ρ*_max_, we match the DC wave functions to asymptotic channel functions corresponding to ro-vibrational levels of the KRb and K_2_ molecules, defined in Jacobi coordinates. This includes vibrational levels up to 2 for KRb and 5 for K_2_ with rotational levels up to a maximum of 68 and 93, respectively.

### Data availability

The data that support the findings of this study are available from the authors on reasonable request.

## Additional information

**How to cite this article:** Croft, J. F. E. *et al*. Universality and chaoticity in ultracold K+KRb chemical reactions. *Nat. Commun.* **8,** 15897 doi: 10.1038/ncomms15897 (2017).

**Publisher’s note:** Springer Nature remains neutral with regard to jurisdictional claims in published maps and institutional affiliations.
